# The effectiveness of a community-based, type 2 diabetes prevention programme on health-related quality of life. The DE-PLAN study

**DOI:** 10.1371/journal.pone.0221467

**Published:** 2019-10-11

**Authors:** Georgios Karamanakos, Bernardo Costa-Pinel, Aleksandra Gilis-Januszewska, Dzilda Velickiene, Francisco Barrio-Torrell, Xavier Cos-Claramunt, Santiago Mestre-Miravet, Beata Piwońska-Solska, Alicja Hubalewska-Dydejczyk, Jaakko Tuomilehto, Stavros Liatis, Konstantinos Makrilakis

**Affiliations:** 1 First Department of Propaedeutic Medicine, Diabetes Center, National and Kapodistrian University of Athens, Laiko General Hospital, Athens, Greece; 2 Catalan Diabetes Prevention Research Group, Jordi Gol Primary Care Research Institute, Catalan Health Institute, Primary Health Care Division, Reus-Barcelona, Spain; 3 Department of Endocrinology, Jagiellonian University, Medical College, Krakow, Poland; 4 Lithuanian University of Health Sciences, Institute of Endocrinology, Kaunas, Lithuania, LT; 5 Department of Public Health Solutions, National Institute for Health and Welfare, Helsinki, Finland; 6 Department of Public Health, University of Helsinki, Helsinki, Finland; 7 Saudi Diabetes Research Group, King Abdulaziz University, Jeddah, Saudi Arabia; German Diabetes Center, Leibniz Center for Diabetes Research at Heinrich Heine University Düsseldorf, GERMANY

## Abstract

**Background and aims:**

The DE-PLAN was a European multicenter study, with the primary objective of testing whether a community-based lifestyle modification programme could serve as a means of primary prevention for type 2 diabetes (T2D) in high-risk individuals (based on the FINDRISC questionnaire). The aim of this study was to examine the impact of a 1-year community-based lifestyle intervention on health-related quality of life (HRQOL) in individuals from four participating European centers (Athens, Barcelona, Krakow, Kaunas), through a post-hoc analysis.

**Materials and methods:**

Each center was allowed to implement different intervention strategies specifically tailored to the needs of their corresponding population sample. Before and after the intervention, participants underwent clinical evaluation, anthropometric measurements, an oral glucose tolerance test and lipid profile measurements. Health-related quality of life was assessed using the validated HRQOL-15D questionnaire. A difference of ±0.015 in the 15D questionnaire score was set as the threshold of clinically meaningful change.

**Results:**

Data from 786 participants (67% females, mean age 59.7±9.4 years, BMI 31.5±4.5 kg/m^2^) with complete data regarding the HRQOL were analyzed (Athens: 104, Barcelona: 434, Krakow: 175, Kaunas: 70). After 1 year, a significant overall improvement in HRQOL was shown, as depicted by a change of 15D score from baseline value (0.88±0.9) to post-intervention (0.90±0.87, P<0.001), achieving the threshold of clinically meaningful change. A significant weight reduction was also observed (-0.8±4.0 kg, P<0.001). In multivariate analysis, improvement in HRQOL was independently associated with lower 15D score at baseline (P<0.001) and self-reported increase in overall exercise time (P<0.001) as assessed through specifically designed trial questionnaires.

**Conclusion:**

A community-based lifestyle intervention programme aiming at T2D prevention, applied on a heterogeneous population and with varied methods, was shown to improve overall health-related quality of life to a clinically meaningful degree.

## Introduction

It has been previously shown that lifestyle modification effectively prevents or delays the development of type 2 diabetes [1], [2], at times with more enduring results than drug therapies [2]. These trials used intense, individually targeted, physical activity and dietary interventions and included people at high risk of developing the disease, i.e. those with impaired glucose tolerance (IGT). While the results from such studies prove the hypothesis that type 2 diabetes is preventable, they were accomplished within a controlled “academic” clinical trial setting, which entails specific inclusion criteria, regular follow-up, intensive interventions and a high conduction cost. In an attempt to translate the effectiveness of the Finnish Diabetes Prevention Study (DPS) [1] and the Diabetes Prevention Program (DPP) [2] through more pragmatic community-based interventions, several studies have been conducted, albeit with significant variance in their corresponding efficacy [3].

A recent systematic review [4] of community-based type 2 diabetes prevention studies questioned their translational value by demonstrating the superiority of the clinical trial setting over more pragmatic interventions, both in terms of weight loss and prevention of type 2 diabetes, highlighting the need of acquiring further information, in order to implement successful and cost-effective type 2 diabetes prevention strategies in public health policy.

In order to assess the feasibility of community-lifestyle interventions to address the need for screening and prevention of type 2 diabetes, a project called “Diabetes in Europe—Prevention using Lifestyle, Physical Activity and Nutritional Intervention” (DE-PLAN) [5] was developed as a European community-based lifestyle intervention study. It aimed at establishing a programme that would be efficient in identifying individuals at high risk of developing type 2 diabetes, as well as testing whether the effectiveness of previously conducted prevention studies could be successfully reproduced and incorporated in the existing healthcare systems. Since type 2 diabetes is a chronic disease with serious complications, imposing a significant burden on the health status of affected individuals, both on physical and mental aspects, it is expected, and indeed well-established, that diabetic patients’ quality of life (total physical, mental, and social well-being) is adversely affected [6]; this may be caused by the impact of the diagnosis of diabetes itself, the psychological stress associated with the management of the disease or the burden of diabetic complications [7]. Several studies in the literature have reported deterioration of the psychological state in people with diabetes and improvement with treatment [8],[9].

Although diabetes prevention studies should—and in fact always do—take into account objective and quantifiable parameters such as weight, plasma glucose and lipid profile, the importance of HRQOL, subjectively evaluated by the participants, has often been overlooked. Clinical and biological variables alone do not comprehensively capture the patients’ perception of their health, which is mainly due to the fact that HRQOL is influenced by multiple factors, such as comorbidities, social and marital status, patient knowledge, treatment satisfaction and perceived ability to control one’s disease [10]. HRQOL is closely connected to how well the person feels and functions. To our knowledge, very limited and controversial data exist in the literature concerning the HRQOL of persons with pre-diabetes and the effects of interventions [11], [12], [13], [14].

The aim of the present study was to examine the impact of a community-based, type 2 diabetes prevention programme, on the HRQOL of the participants, through the analysis of the DE-PLAN data.

## Design and methods

The DE-PLAN study protocol has been previously described in detail [5]. In brief, in order to identify individuals at high risk of developing diabetes, community screening was conducted with the use of the validated Finnish type 2 Diabetes Risk Score (FINDRISC) questionnaire [15]. An oral glucose tolerance test (OGTT) was used on high risk individuals (those with FINDRISC >14, having an estimated 50% probability of developing diabetes within the succeeding 10 years) in order to exclude those with previously unknown diabetes. Eligible participants were then enrolled in a country/center specific community-based lifestyle intervention programme, aiming at weight loss, reduced intake of total and saturated fat, increased consumption of dietary fiber and increased physical activity.

The assessment of the impact of the intervention on the participants’ HRQOL was also part of the DE-PLAN study protocol. The protocol was approved by the corresponding ethics committees (**Athens**: Laiko General Hospital Ethics Review Board 1/2/2007 ref No:1067, **Barcelona**: Jordi Gol Research Institute ref No:P05/38/2006, **Krakow**: Jagiellonian University Ethics Committee, ref No: KBET/43/L/2006, **Kaunas**: Regional Bioethics Committee, Lithuania ref No:36/2006) and all participants signed an informed consent in accordance with the Good Clinical practice rules outlined in the declaration of Helsinki [16].

An aggregate report of the data acquired during the DE-PLAN study has not been published yet. However, some individual participating countries have reported the results of the locally implemented lifestyle interventions [17], [18], [19], [20]. In the present study, the data related to HRQOL from the four European countries/centers (Greece/Athens [17], Spain/Barcelona [18], Poland/Krakow [19] and Lithuania/Kaunas) with complete relevant data from the DE-PLAN study were analyzed.

The initial DE-PLAN study was not registered in a clinical trial registry, since this was not compulsory at that time. Registrations were carried out retrospectively for some centers at a later stage, after the beginning of recruitment. Accordingly, the present study does not have a registration number for all participating centers. Registration of the remaining centers retrospectively is under way.

### Screening and enrolment

**Fig 1:**
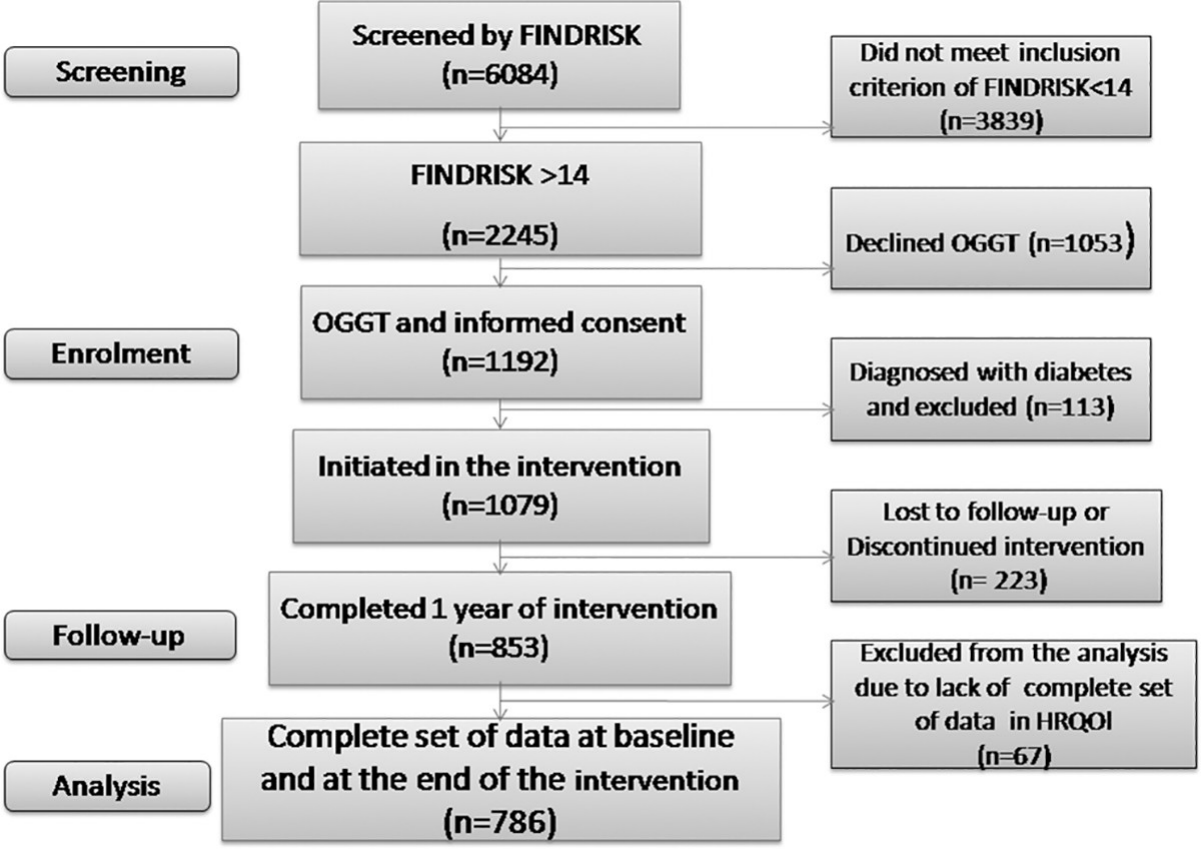
Flow chart depicting the size of the cohort of participants during the successive steps of the study, from screening to completion. FINDRISC: Finnish type 2 diabetes risk score, OGTT: Oral glucose tolerance test.

The DE-PLAN study was conducted from 2005 to 2009. In the primary screening phase of the study, 6,084 participants filled-in the FINDRISC questionnaire. A total of 2,245 individuals with a score >14 were identified and subsequently invited to undertake an oral glucose tolerance test (OGTT); 1,192 of them agreed to undertake the test and sign the informed consent. After excluding those who were diagnosed with diabetes during the OGTT, 1,079 individuals were enrolled in the 1-year interventional part of the study. A complete assessment, including a second OGTT, was performed at the end of the intervention on 853 (79%) participants of the initial cohort. A complete case data analysis was performed on 786 individuals with a complete set of data regarding HRQOL, at both the beginning and the end of the intervention (Fig 1)

During the first OGTT appointment, a detailed medical and lifestyle history was obtained using questionnaires (FINDRISC, Basic and Quality of Life questionnaires; see Supplementary material) provided by the DE-PLAN study protocol [5]. The questionnaires used for the basic information were created for this specific study based on the principles of the DPS [1]. Although they had not been previously validated, they used an easy to understand language and offered the participants a way to self-evaluate their level of socioeconomic and physical activity status. Specifically, participants answered questions concerning their physical activity habits over the previous year (reported information on occupation and leisure-related daily physical activity), as well as a combined self-assessment overall exercise time (low, medium, high, and very high). Socioeconomic factors such as marital status, occupation and educational level (basic educational training: 9 years of school, intermediate: 12 years of school, and high: university and above) were also reported. Anthropometric measurements such as height, weight (with minimal clothing) and waist circumference (at the midpoint between iliac crest and costal arch) were recorded by trained study personnel. Blood pressure was measured by physicians, twice, 2 min apart, in a sitting position, and the average of the two measurements was calculated and reported. Plasma glucose (fasting and 2 hours after the OGTT), total and high-density lipoprotein cholesterol (HDL-C) and triglyceride levels were measured in a central laboratory at each center. Low-density lipoprotein cholesterol (LDL-C) was calculated using the Friedewald formula [21]. Diabetes was diagnosed using the WHO [22] definition (either fasting plasma glucose ≥126 mg/dl (7.0 mmol/l], or a 2–h plasma glucose ≥200 mg/dl [11.1 mmol/l]). Pre-diabetes was defined as either impaired fasting glucose (plasma glucose in the morning, after an 8-h fast higher than 99 mg/dl [5.5 mmol/l] but lower than 126 mg/dl [7.0 mmol/l]) or impaired glucose tolerance (plasma glucose 2 hours after a 75 g oral glucose load higher than 139 mg/dl [7.7 mmol/l]) but lower than 200 mg/dl [11.1 mmol/l]).

### Health related quality of life assessment

The participants’ HRQOL was evaluated using the 15D questionnaire [23]. This is a generic, self-rated tool of HRQOL assessment that has been externally validated and is of similar predictive value as other widely used questionnaires [24]. It is comprised of 15 dimensions (questions) of functionality, namely: mobility, vision, hearing, breathing, sleeping, eating, speech, excretion, usual activities, mental function, discomfort and symptoms, depression, distress, vitality and sexual activity, each having five different levels of functional status for each individual parameter, arranged in an ordinal manner, with the higher order corresponding to higher degree of disability. These dimensions can be reported both individually or as a one-index score (D15 score), by using utility weights based on an application of the multi-attribute utility theory. The single index score ranges from zero (0) HRQOL (essentially dead), to 1 corresponding to optimal self-assessed HRQOL. A recent study [25] by Alanne *et al*, conducted on 4,903 hospitalized patients, concluded that a change of ±0.015 of the 15D score is considered as the Minimum Important Difference and represents a clinically meaningful change.

### Intervention

Taking into account the high diversity between existing European healthcare systems and the cultural-ethnic multiplicity in Europe, it is evident that a generic, one-size-fits-all approach would be impractical. The DE-PLAN study protocol [5] allowed each country to form its own intervention strategy, with the objective of educating participants on the modifiable risk factors for the development of diabetes, as well as by offering them the means to achieve enduring lifestyle changes. Within two weeks after the end of the lifestyle intervention, a final (follow-up) visit was arranged for overall assessment of the participants, including virtually all the parameters recorded during the baseline visit. A brief description of each lifestyle intervention protocol used by the four countries participating in the present analysis is as follows:

#### Athens (Greece)

Based on the five prevention goals set forth by the Finnish DPS study [26], participants in the Greek arm [27] of the DE-PLAN were offered six group (comprised of 6–10 persons) hourly sessions with a qualified dietitian, near the area of their work or residence. In these sessions, through individual and group discussions and written supplements, an effort was made to increase awareness on the risk of developing diabetes and building up motivation for change. Additionally, behavioral empowerment was considered with the provision of dietary recommendations and meal planning for a healthier lifestyle. While there were no formal exercise sessions, participants were strongly encouraged to incorporate more physical activity in their everyday life.

#### Barcelona (Catalonia, Spain)

Participants allocated to the intensive lifestyle intervention arm were offered a total of six hours of educational sessions, depending on the corresponding center resources, either in individual or in group sessions of 5–15 persons. These consultations aimed at familiarizing participants with the risk of developing the disease and at highlighting the importance of increased exercise frequency, smoking cessation and the Mediterranean diet as tools of diabetes prevention. Specifically, for nutritional advice and diet adherence improvement the “Prevención con Dieta Mediterránea-Mediterranean Diet Adherence Screener” (PREDIMED-MEDAS) questionnaire was used [28]. Finally, a regular contact strategy every 6–8 weeks, either by phone calls or text messaging, was applied to maintain motivation. In Catalonia the modified DE-PLAN intervention has been implemented as a population strategy [29].

#### Krakow (Poland)

The Polish arm of the DE-PLAN study [19],[30],[31],[32] established an intervention following the steps of the DPS [26], modified and adjusted to a local primary healthcare setting. Nurses (two per center), specifically-trained and certified in diabetes prevention, delivered a 10-month intervention of behavioral modification, focusing on dietary advice for the adoption of healthier eating patterns and increase of physical activity. The initial intensive phase of the intervention lasted four months and consisted of one individual session followed by ten group sessions of 10 to 14 people. During each session, printed educational materials related to the topic of the session were distributed. Social support was emphasized by the group setting and participants were also encouraged to invite members of their own social environment to implement lifestyle changes. A spouse or other family member could also participate in the sessions. From week four of the intervention initiation, participants were offered physical activity sessions free of charge (aqua aerobics and gymnastics or football) twice a week. The maintenance phase of the intervention (month 4–10) consisted of six motivational telephone calls and two motivational letters.

#### Kaunas (Lithuania)

Participants from Lithuania were invited to participate in an intervention that was delivered by an endocrinologist and a nurse specialized in diabetes education. On the initial individual consultation, specific information and treatment-tailored tasks were given by the medical personnel. Subsequently, group sessions of 6–8 people focusing on diet, and benefits of physical activity were performed every two weeks for the first two months, once a month for the following four months and every three months for the final six months. Participants were also offered phone consultations by the study staff, free of charge, at any stage during the intervention. Printed educational material was provided to all participants at all sessions. The participants were also able to share their experience within the group, measure body weight, waist circumference, and give anonymous feedback. Spouse and family member attendance was strongly encouraged. All participants were offered two weekly physical activity sessions at the gym, at a 50% reduced fee, under the supervision of an experienced coach. The sessions’ schedule was modified according to the participant's needs and timetable, and a reminder phone call was given before each training session.

### Statistical analysis

A complete case analysis of individuals with a complete set of answers on all the dimensions of the HRQOL-D15 questionnaire, both at baseline and at 1 year after the intervention was performed. In order to account for attrition bias, baseline characteristics of participants with partly filled questionnaires, i.e. whose D15 score could not be computed (for any reason), were compared to those of analyzed cases. Anthropometric, laboratory, socioeconomic and physical activity parameters were analyzed in order to assess for potential correlations with HRQOL. The change/improvement of D15, calculated by the difference between baseline and follow-up values, was used as the dependent variable for the final measure of HRQOL.

A univariate analysis of factors that have been commonly associated with HRQOL was conducted in order to examine their effect on the change of HRQOL. Variables that exhibited a significant association in the univariate analysis, as well as the baseline glucose values (for reasons of good practice) and the variable "center” (in order to control for differences between interventions and population heterogeneity), were introduced in a multiple linear regression model. The backward stepwise method was used to determine the parameters with independent significance. The P value that was used for introduction into the model was 0.1.

The potential effects of the intervention in people with prediabetes was examined through a subgroup analysis on their respective weight and HRQOL changes.

Comparisons between two variables with normally distributed mean values were performed by the independent Student’s t-test (for independent observations) or the paired sample t-test (for paired observations). For comparisons between non-normally distributed data, the Mann-Whitney test (for independent observations) and the related samples Wilcoxon signed-rank test (for paired observations) were used.

Data were analyzed using the SPSS version 20.0 (SPSS Inc., Chicago, IL, USA). A P-value equal or less than 0.05 was considered statistically significant.

## Results

The final study population included 786 individuals from four European cities (Athens: n = 107, Barcelona: n = 434, Krakow: n = 175, and Kaunas n = 70). Baseline characteristics of the participants are shown in Table 1. The majority were female (67.0%) and of Catalonian origin (55.2%). Pre-diabetes was identified in 44.5% of the cohort. The participants’ mean BMI was high (31.5±4.5 kg/m^2^), with a mean weight of 82.7±14.3 kg and a mean waist circumference of 104.5±9.5 cm and 98.4±10.4 cm, for men and women, respectively. Mean values of baseline HRQOL measurements, as described by the combined D15 index score, were somewhat different among the study centers (Table 2), with participants from Athens (0.89±0.08) and Barcelona (0.89±0.09) having similar values that were higher than the respective ones from Krakow (0.87±0.09) and Kaunas (0.84±0.10). Overall, a significant improvement in HRQOL was observed at the end of the 1-year intervention, as exhibited by positive changes in the D15 score (P<0.001). The net mean difference between baseline and follow-up values of D15 score was 0.015, reaching the proposed threshold level [25] of Minimum Important Difference. Regarding the individual dimensions of the HRQOL in the D15 questionnaire (Table 3), significant gains in functionality were noted in the comparison of baseline to follow-up values in the following categories: breathing (P = 0.01), sleeping (P<0.001), excretion (P<0.001), usual activities (P = 0.02), mental function (P<0.001), discomfort and symptoms (P<0.001), depression (P<0.001), distress (P<0.001) and vitality (P<0.001).

**Table 1:** Baseline characteristics of participants (mean ±SD).

Variable	Athens	Barcelona	Kaunas	Krakow	Total
Total number of participants	107	434	70	175	786
Male/Female (n, %)	52 (48.6%) /55 (51.4%)	153 (35.3%) /281 (64.7%)	16 (22.9%) /54 (77.1%)	38 (21.7%) /137 (78.3%)	259 (33%) /527 (67%)
Age (years)	54.5 ±9.9	62.2 ±7.7	61.7 ±7.5	56.1 ±10.9	59.7 ±9.4
Current smokers (n, %)	58 (54.7%)	164 (37.8%)	22 (31.4%)	84 (48%)	328 (41.7%)
Weight (kg)	89.2 ±13.5	78.8 ±11.9	89.9 ±13.1	85.6 ±16.1	82.7 ±14.3
BMI (kg/m^2^)	31.8 ±3.9	31.2 ±4.4	32.9 ±4.47	31.7 ±5.0	31.5 ±4.5
Participants with pre-diabetes (n, %)	74 (69.1%)	189 (43.5%)	42 (60%)	45 (25.6%)	350 (44.5%)
Waist circumference (cm)	102.6 ±10.8	100.6 ±10	100.7 ±9.6	98.7 ±11.1	100.4 ±10.5
Male (n = 259)	108.1 ±9.7	102.9 ±8.6	105.6 ±7.4	106.1 ±12	104. 5 ±9.5
Female (n = 527)	97.4 ±91	99.3 ±10.4	99.3 ±9.8	96.7 ±10.9	98.4 ±10.4
Systolic blood pressure (mmHg)	132.7 ±17.5	136.5 ±16.3	146.8 ±18.4	133.2 ±15.7	136.2 ±16.9
Diastolic blood pressure (mmHg)	80.1 ±13.5	80.4 ±9.6	87.8 ±9.4	83 ±9.4	81.6 ±10.4
Fasting Glucose (mmol/l)	5.8 ±0.6	5.2 ±0.7	6.1±0.6	5.2 ±0.7	5.4 ±0.7
Total cholesterol (mmol/l)	5.8 ±1.08	5.4 ±0.9	6.2 ±1.2	5.5 ±1	5.5 ±1
HDL cholesterol (mmol/l)	1.3 ±0.2	1.4 ±0.4	1.3 ±0.3	1.4 ±0.4	1.4 ±0.35
Triglycerides (mmol/l)	1.4 ±0.9	1.4 ±0.8	2.1 ±0.8	1.7 ±1.2	1.5 ±0.9
LDL cholesterol (mmol/l)	3.9 ±0.9	3.3 ±0.9	3.8 ±1.1	3.4 ±1	3.4 ±0.93

Mean ±SD or n (%). BMI, body mass index; HDL, high-density lipoprotein; LDL, low-density lipoprotein

**Table 2:** Baseline and post-intervention values of D15.

Center	D15 score at baseline	D15 score post intervention	Difference	P-value
Athens	0.89 ±0.08	0.91 ±0.08	0.02	0.136
Barcelona	0.89 ±0.09	0.90 ±0.09	0.01	0.246
Kaunas	0.84 ±0.10	0.90 ±0.09	0.06	**<0.001**
Krakow	0.87 ±0.09	0.90 ±0.09	0.03	**<0.001**
Combined sample	0.88 ±0.09	0.90 ±0.09	0.02	**<0.001**

**Table 3:** Changes in D15 score and its individual dimensions between baseline and post-intervention (mean±SD).

Variable	Baseline value	Post intervention	P-value
D15 score	0.88 ±0.9	0.90 ±0.87	**<0.001**
Mobility	0.915 ±0.14	0.919 ±0.14	0.6
Vision	0.885 ±0.17	0.884 ±0.17	0.6
Hearing	0.941 ±0.12	0.948 ±0.12	0.1
Breathing	0.87 ±18	0.89 ±17.8	**0.01**
Sleeping	0.78 ±0.2	0.81 ±0.2	**<0.001**
Eating	0.99 ±0.36	0.99 ±038	0.9
Speech	0.98 ±0.66	0.98 ±0.67	0.7
Excretion	0.88 ±0.17	0.91 ±0.15	**<0.001**
Usual activities	0.90 ±0.16	0.91 ±0.15	**0.02**
Mental function	0.87 ±0.16	0.90 ±0.15	**<0.001**
Discomfort and symptoms	0.83 ±0.18	0.86 ±0.17	**<0.001**
Depression	0.83 ±0.19	0.85 ±0.18	**<0.001**
Distress	0.77 ±0.18	0.80 ±0.18	**<0.001**
Vitality	0.81 ±0.17	0.83 ±0.17	**<0.001**
Sexual activity	0.83 ±23	0.82 ±22	0.1

Several favorable changes in anthropometric and clinical measures were observed after the intervention (Table 4). Significant weight reduction was achieved in the study sample (median: 0.5 kg, interquartile range from 3.0 kg reduction to 1.45 kg gain, P<0.001, mean: 0.83 ±4.0 kg, P<0.001), albeit with substantial differences among individual centers: a mean of -0.7±4.7 kg reduction in Athens, -0.2 ±3.9 kg in Barcelona, -1.9±5.0 kg in Krakow and -2.0±3.4 kg in Kaunas. A slightly higher mean weight loss was observed among individuals identified to have pre-diabetes at baseline (0.93±4.1 kg) compared with the normoglycemic people (0.76±4.5 kg, P<0.001). Improvements were also noted in waist circumference, blood pressure, and lipid profile of the participants. The percentage of individuals with pre-diabetes was substantially reduced at the end of the intervention, from 44.5% (350/786) to 34% (267/786), with a simultaneous increase of the normoglycemic population from 51.5% (436/786) to 61.2% (481/786). A total of 35 (4.4%) participants developed diabetes by the end of the programme.

**Table 4:** Changes in anthropometric and clinical parameters at baseline and post-intervention assessments (mean ±SD).

Characteristic	Baseline value	Post intervention	Difference*	P-value
Weight (kg)	82.7 ±14.3	81.9 ±13.9	0.8 ±4.0	<0.001
BMI (kg/m^2^)	31.5 ±4.5	31.2 ±4.6	0.3 ±1.7	<0.001
Waist circumference (cm)	100.4 ±10.5	99.7 ±10.8	0.7 ±6.5	0.01
Systolic blood pressure (mmHg)	136.2 ±16.9	133.6 ±16.4	2.6 ±16.5	<0.001
Diastolic blood pressure (mmHg)	81.6 ±10.3	79.3 ±9.5	2.6 ±10.9	<0.001
Fasting Glucose (mmol/l)	5.4 ±0.7	5.4 ±0.9	0	0.28
Total cholesterol (mmol/l)	5.5 ±1	5.4 ±1.1	0.2 ±1.9	<0.001
HDL cholesterol (mmol/l)	1.4 ±0.35	1.36 ±0.34	0.1 ±0.3	<0.001
Triglycerides (mmol/l)	1.5 ±0.9	1.5 ±0.9	0.04 ±0.8	0.18
LDL cholesterol (mmol/l)	3.4 ±0.93	3.3 ±1.1	0.1 ±1	<0.001

* Difference refers to “difference of baseline minus post-intervention value”

BMI, body mass index; HDL, high-density lipoprotein; LDL, low-density lipoprotein

Physical activity parameters improved at the end of the intervention compared with the baseline, as indicated by the duration of exercise time during work (P = 0.01) and leisure time (P<0.001), self-assessed physical state (P<0.001), and overall increase in exercise time over the last year (P<0.001).

In order to explore the mediators of 15D improvements between baseline and 1-year follow-up, a univariable analysis (Table 5) of factors that were considered to play a role in such a change was conducted. The 15D improvement significantly predicted the amount of weight loss (P = 0.04), self-reported intensification of overall exercise time (P<0.001), female sex (P = 0.01), higher educational level (P = 0.001), lower baseline 15D score (P<0.001) and the study center (Kaunas > Krakow > Athens > Barcelona) (P<0.001). A trend was also noted between 15D improvement and the presence of pre-diabetes at baseline (P = 0.08), while no association was noted with age, BMI at baseline, indices of physical activity at baseline and marital status.

**Table 5:** Univariable analysis examining the predictors of D15 score improvement.

Variable	Standardized Beta Coefficients	Univariable analysis (P-value)
Weight loss	0.069	**0.04**
Increase of overall exercise time	0.159	**<0.001**
Female gender	0.088	**0.014**
Educational level	0.105	**0.001**
Baseline 15D score	-0.441	**<0.001**
Presence of pre-diabetes at baseline	-0.056	**0.08**
Age	0.020	0.9
BMI at baseline	0.034	0.8
Overall physical activity at baseline	-0.053	0.1
Marital status	0.074	0.1

In order to control the associations’ effects of covariates, all of the possible mediators of HRQOL improvement were inserted in a multiple linear regression model. Participants’ age and baseline glucose value were also introduced in the model for reasons of good practice, despite their lack of statistically significant association in the univariable analysis. In the final multivariable model (Table 6**)**, improvement in D15 score was independently associated with a lower D15 score at baseline (P<0.001), the conduction of the study at the center in Kaunas (P = 0.049), which was the only study center that retained statistical significance in the multivariate model when compared to the baseline center (Barcelona), and with high and very high self-reported increase in overall physical activity time during the intervention programme (P<0.001).

**Table 6:** Multivariable linear regression analysis examining the mediators of D15 score improvement.

Variable	Standardized Beta Coefficients	Multivariate analysis (P-value)
Baseline D15 score	-0.444	**<0.001**
Center-combined	0.119	**<0.001**
Center^1^-Athens	0.005	0.8
Center^1^ -Krakow	0.066	0.1
Center^1^ -Kaunas	0.090	**0.049**
Age	-0.026	0.4
Gender (male/female)	-0.041	0.2
Weight loss	0.039	0.2
Educational level-combined	0.026	0.5
Increased physical activity time over the past year-combined	0.157	**<0.001**
Increased physical activity time over the past year^2^ -Minimal improvement	-0.003	0.9
Increased physical activity time over the past year^2^-Moderate improvement	0.019	0.6
Increased physical activity time over the past year^2^-High improvement	0.137	**<0.001**
Increased physical activity time over the past year^2^ -Very high improvement	0.125	**0.001**
Baseline glucose value	0.014	0.6

1: The center of Barcelona [i.e. the center with the greatest number of participants (55.2% of the analyzed sample)] was selected as the reference center for comparisons between centers

2: Absence of improvement in exercise time over the past year was selected as the reference for comparison of exercise improvements.

## Discussion

In the present analysis the effect on HRQOL of a real-life lifestyle intervention programme aiming to prevent type 2 diabetes, conducted in four European countries, is reported. It was shown that significant improvements were attained after 1-year of intervention in overall HRQOL, as measured by the validated D15 questionnaire [23], as well as in individual dimensions of the score. Further on, the level of change in HRQOL achieved the recently proposed level of minimum clinical reference, that was +0.015 [25].

While several lifestyle intervention studies have been conducted since the landmark DPS [1] and DPP [2] studies, data regarding HRQOL have been scarce in such studies. The DPP investigators reported that during the first year of intervention, physical function, general health and vitality scores improved significantly in the intensive lifestyle intervention arm as compared to the placebo and metformin arms [33]. In the community setting, a small single center study in Malaysia has reported that HRQOL improved after a one-year lifestyle intervention in individuals with pre-diabetes [34], while in the Australian Greater Green Triangle Diabetes Prevention Project [35], significant improvements in several psychological measures were observed after a one-year community based intervention in participants with pre-diabetes.

According to the present analysis, the main factors independently associated with improvement in HRQOL were a lower self-reported HRQOL at baseline, the self-reported increase in physical activity time during the study and with the conduction of the study in the center of Kaunas. Results from both RCTs [36] and observational studies [37] support the notion that the maximum change in HRQOL is observed in individuals with greater impairment in quality of life at baseline. This is particularly important since, in a recent study, low HRQOL measured by 15D was associated with unhealthier lifestyle choices and more symptoms of depression [38]. The participants’ metabolic and anthropometric parameters (glucose, blood pressure and BMI), that are indicative of a low level of health, were found to be increased, as expected for people at increased risk for development of type 2 diabetes [39]. Presumably, any gains in HRQOL in such individuals not only may achieve immediate benefits of improved functionality, but also lead to successful behavioral modification. This in turn can result in healthier adjustments in everyday life that are sustained after the end of the intervention programmes. It is worth noting that in the present study, the most significant improvements out of the individual 15D-score dimensions were observed in psychological parameters, such as sleeping, distress and depression.

General population studies [40] have established that increased levels of physical activity/exercise are consistently correlated with improved HRQOL, while the exact reason of such an association remains unclear. Specifically for individuals with pre-diabetes, the analysis of changes in HRQOL [33] during the DPP study also reported an association with advances in physical activity levels at the end of the lifestyle intervention. This association however was considered partially mediated through weight reduction that to some extent is a result of energy expenditure due to physical activity. In contrast, the present study results showed that an increase in overall exercise time was one of the main predictors of HRQOL improvements, even when controlling for weight loss. This finding was rather unexpected given the fact that both the Greek and the Catalonian centers did not include a specific dedicated exercise programme as part of their lifestyle intervention strategies. It appears that improvements in overall time of physical activity in the present study might also have been attributed to other factors of the intervention, such as increased motivation and improved knowledge gained in the counseling sessions, as well as the support and encouragement that was provided by study personnel during regular contact. On the other hand, the lack of a significant independent association between HRQOL improvement and weight loss may be attributed to the magnitude of weight reduction achieved, which was small in the present study. Furthermore, although weight loss is certainly important and was considered to be the main mediator of HRQOL improvement in the DPP study [33], results from a systematic review [41] did not report a consistent association between these two parameters.

When the different centers were inserted into the multivariable regression model as independent covariates, it was shown that the conduction of the study in Kaunas was associated with higher HRQOL improvement. Reasons for that can only be speculated, but two of the key differences that can be identified were the higher intensity of the programme and the worse baseline metabolic profile of participants in Kaunas. Specifically, participants in Kaunas had the highest baseline BMI, blood glucose and arterial blood pressure values among the study centers (Table 1). It is therefore possible that the intervention resulted in a greater magnitude of change in those more metabolically compromised individuals, in turn resulting in higher improvements in D15 score. The higher intensity of the programme is also evident since both the Polish and Lithuanian centers included a structured exercise regime in their intervention, which may have been a key difference compared with Greece and Catalonia, where only encouragement and educational tactics to indirectly increase physical activity were used. Notably, weight reduction was greatest in the Polish and Lithuanian centers (1.9 and 2 kg, respectively).

Further-on, the independent correlation trend between the conduction center and HRQOL improvement, irrespective of weight loss and overall physical activity gains, suggests that additional factors are implicated. For example, there were differences between intervention centers in the personnel instructing the consultations, with centers in Krakow and Kaunas using specifically trained diabetes nurses, while certified dietitians were used in Athens. The number of consultations also differed between centers, with Athens and Barcelona using six sessions and the Polish and Lithuanian centers eleven and ten sessions, respectively. All these factors, coupled with the ethnical-cultural variation, may have contributed to the different functionality gains observed.

According to the present study findings, the change in 15D after the intervention attained a clinically meaningful minimum important difference level of 0.015 [25]. Recently, data regarding the actual clinical significance of HRQOL have been brought to light. In a large quality of life sub-study by Hayes et al. [42], based on the Fenofibrate Intervention and Event Lowering in Diabetes (FIELD) study [43] population, better HRQOL was associated with significantly less risk for vascular events and other diabetic complications such as heart failure, end-stage renal disease and leg ulcers. Further-on, a recent prospective cohort study [44] highlighted the association between poor self-reported HRQOL and mortality in patients with diabetes. Alarmingly, population studies [14] have shown that the level of HRQOL of individuals with pre-diabetes is compromised, ranging between that of NGT and diabetes [45]. A population study in Finland showed an inverse association between self-reported HRQOL and the FINDRISC score [46]. Given the link between HRQOL and mortality, the fact that several individuals with newly diagnosed diabetes already present with chronic disease-associated microvascular complications [47],[48] and that patients with type 2 diabetes and people with pre-diabetes have increased all-cause, cardiovascular and cancer mortality compared with those with normoglycemia [49],[50],[51],[52], it becomes evident that improvements in self-perceived health status and HRQOL should be a main target of interventions aiming at preventing diabetes.

The main strengths of the present study include the multicenter and multinational design, the use of an externally validated diabetes risk questionnaire [23] coupled with an OGTT, both at baseline and at the end of the intervention, and the fact that all study procedures, including anthropometric measurements and questionnaire assessments were made by health care personnel trained for the study. Most importantly, the study was performed in a community-based setting, similar to real world circumstances.

The statistical method that was selected for our analysis carries both strengths and weeknesses. A backward stepwise multiple linear regression was used for multivariable analysis, with values retaining statistical significance in the final model considered of having independent predictive value. The backward stepwise method has the advantage of removing generally non-significant predictors, creating a simple final model with the higher correlated variables at the end. However it often also carries the disadvantage of resulting in models that overestimate the magnitude of significance of the retained predictors, with relatively higher R^2^ values in the subsequent models.

The main limitations include the heterogeneity of the intervention strategies and the lack of a control group. Without a comparator control group, changes in HRQOL of participants cannot be definitely attributed to the intervention. Other factors such as the performance bias, i.e. the process in which a person enrolled in a study behaves differently and experiences improvements due to the sense of being part of a study in itself, as well as changes/improvements that may simply be attributed to the passage of time, should be considered.

The original DE-PLAN study [5] protocol was specifically created to test whether a pragmatic community-based diabetes prevention programme could be successfully implemented across the existing and significantly diverse health care systems in Europe, and not to test the effectiveness of such a programme, as this had already been proven in the past by large controlled studies [1],[2]. Thus, a control group was not used in the original DE-PLAN study, which, on the other hand, was purposely flexible regarding implementation, in order to accommodate to national differences in health care structures and resources and to simulate real-life conditions. The participants in our study were volunteers and, like many other such studies, it predominantly attracted women. Thus, our results might not be generalizable to both sexes. In addition, the rather modest weight loss results obtained might be influenced by female sex domination, whose success in previous diabetes prevention studies was meager when compared to men [53]. On the other hand, the preventive effect of lifestyle intervention in the DPS did not differ between men and women [54].

Another limitation of our study is the possible attrition bias. The participants that were originally enrolled but did not complete either the initial or the final evaluation of HRQOL had some significant differences when compared with the ones that had a full set of data. Specifically they were of younger age, had lower baseline systolic blood pressure measurement and a significantly lower baseline HRQOL level (S1 Table). Although we cannot exclude the possibility that people with lower HRQOL might have faced greater difficulty attaining, following and completing a lifestyle intervention programme, the observed difference at baseline was of rather minor clinical magnitude [25] to produce such an effect. On the other hand it is also probable that improved strategies were needed, such as more regular contact in-between visits and the introduction of individual exercise/educational sessions, in order to account for the specific characteristics and challenges associated with participants of lower HRQOL. This could in turn reduce the dropout rate, an important goal, since according to our results, lower D15 at baseline was associated with greater HRQOL gains from the intervention.

A final limitation was the use of non-validated questionnaires for the assessment of physical activity time. Therefore, the finding of increased physical activity time as an independent predictor of D15 change should be viewed with some caution.

In conclusion, the present study demonstrated that a community-based type 2 diabetes prevention programme based on lifestyle intervention may lead to substantial improvements in HRQOL. Since the intervention was implemented on diverse populations and through different strategies, these findings may serve as a realistic perspective of tackling diabetes epidemic across Europe.

## Supporting information

S1 File Supplementary material. (ZIP)

S2 File TREND checklist. (PDF)

S1 Table Supplementary material table: Comparison of baseline characteristics of participants with full set of data vs. participants without. (DOC)
