# Supplementary material for: The effectiveness of a community-based, type 2 diabetes prevention programme on health-related quality of life. The DE-PLAN study
Source: PLoS One. 2019 Oct 11;14(10):e0221467. doi: 10.1371/journal.pone.0221467 (PMC6788719; doi:10.1371/journal.pone.0221467)
Supplement: S1 File — (ZIP) [file pone.0221467.s001.zip › 15DENG-Quality of Life Questionnaire.pdf]

DE-PLAN **Quality of Life Questionnaire (15D©)** **Date of visit (e.g. 260106)**

d d m m y y

Name: ____________________

Identity number: | | | | | | | | | | |

Date of birth (e.g. 231055)

Sex **1 Male**

2 Female

d d m m y y

Please read through all the alternative responses to each question before placing a cross (x) against the alternative which best describes **your present health status**. Continue through all 15 questions in this manner, giving only **one** answer to each.

QUESTION 1. MOBILITY

1 ( ) I am able to walk normally (without difficulty) indoors, outdoors and on stairs.
2 ( ) I am able to walk without difficulty indoors, but outdoors and/or on stairs I have slight difficulties.
3 ( ) I am able to walk without help indoors (with or without an appliance), but outdoors and/or on stairs only with considerable difficulty or with help from others.
4 ( ) I am able to walk indoors only with help from others.
5 ( ) I am completely bed-ridden and unable to move about.

QUESTION 2. VISION

1 ( ) I see normally, i.e. I can read newspapers and TV text without difficulty (with or without glasses).
2 ( ) I can read papers and/or TV text with slight difficulty (with or without glasses).
3 ( ) I can read papers and/or TV text with considerable difficulty (with or without glasses).
4 ( ) I cannot read papers or TV text either with glasses or without, but I can see enough to walk about without guidance.
5 ( ) I cannot see enough to walk about without a guide, i.e. I am almost or completely blind.

QUESTION 3. HEARING

1 ( ) I can hear normally, i.e. normal speech (with or without a hearing aid).
2 ( ) I hear normal speech with a little difficulty.
3 ( ) I hear normal speech with considerable difficulty; in conversation I need voices to be louder than normal.
4 ( ) I hear even loud voices poorly; I am almost deaf.
5 ( ) I am completely deaf.

QUESTION 4. BREATHING

1 ( ) I am able to breathe normally, i.e. with no shortness of breath or other breathing difficulty.
2 ( ) I have shortness of breath during heavy work or sports, or when walking briskly on flat ground or slightly uphill.
3 ( ) I have shortness of breath when walking on flat ground at the same speed as others my age.
4 ( ) I get shortness of breath even after light activity, e.g. washing or dressing myself.
5 ( ) I have breathing difficulties almost all the time, even when resting.

QUESTION 5. SLEEPING

1 ( ) I am able to sleep normally, i.e. I have no problems with sleeping.
2 ( ) I have slight problems with sleeping, e.g. difficulty in falling asleep, or sometimes waking at night.
3 ( ) I have moderate problems with sleeping, e.g. disturbed sleep, or feeling I have not slept enough.
4 ( ) I have great problems with sleeping, e.g. having to use sleeping pills often or routinely, or usually waking at night and/or too early in the morning.
5 ( ) I suffer severe sleeplessness, e.g. sleep is almost impossible even with full use of sleeping pills, or staying awake most of the night.

QUESTION 6. EATING

1 ( ) I am able to eat normally, i.e. with no help from others.
2 ( ) I am able to eat by myself with minor difficulty (e.g. slowly, clumsily, shakily, or with special appliances).
3 ( ) I need some help from another person in eating.
4 ( ) I am unable to eat by myself at all, so I must be fed by another person.
5 ( ) I am unable to eat at all, so I am fed either by tube or intravenously.

QUESTION 7. SPEECH

1 ( ) I am able to speak normally, i.e. clearly, audibly and fluently.
2 ( ) I have slight speech difficulties, e.g. occasional fumbling for words, mumbling, or changes of pitch.
3 ( ) I can make myself understood, but my speech is e.g. disjointed, faltering, stuttering or stammering.
4 ( ) Most people have great difficulty understanding my speech.
5 ( ) I can only make myself understood by gestures.

QUESTION 8. ELIMINATION

1 ( ) My bladder and bowel work normally and without problems.
2 ( ) I have slight problems with my bladder and/or bowel function, e.g. difficulties with urination, or loose or hard bowels.
3 ( ) I have marked problems with my bladder and/or bowel function, e.g. occasional 'accidents', or severe constipation or diarrhea.
4 ( ) I have serious problems with my bladder and/or bowel function, e.g. routine 'accidents', or need of catheterization or enemas.
5 ( ) I have no control over my bladder and/or bowel function.

QUESTION 9. USUAL ACTIVITIES

1 ( ) I am able to perform my usual activities (e.g. employment, studying, housework, free-time activities) without difficulty.
2 ( ) I am able to perform my usual activities slightly less effectively or with minor difficulty.
3 ( ) I am able to perform my usual activities much less effectively, with considerable difficulty, or not completely.
4 ( ) I can only manage a small proportion of my previously usual activities.
5 ( ) I am unable to manage any of my previously usual activities.

QUESTION 10. MENTAL FUNCTION
1 ( ) I am able to think clearly and logically, and my memory functions well
2 ( ) I have slight difficulties in thinking clearly and logically, or my memory sometimes fails me.
3 ( ) I have marked difficulties in thinking clearly and logically, or my memory is somewhat impaired.
4 ( ) I have great difficulties in thinking clearly and logically, or my memory is seriously impaired.
5 ( ) I am permanently confused and disoriented in place and time.

QUESTION 11. DISCOMFORT AND SYMPTOMS
1 ( ) I have no physical discomfort or symptoms, e.g. pain, ache, nausea, itching etc.
2 ( ) I have mild physical discomfort or symptoms, e.g. pain, ache, nausea, itching etc.
3 ( ) I have marked physical discomfort or symptoms, e.g. pain, ache, nausea, itching etc.
4 ( ) I have severe physical discomfort or symptoms, e.g. pain, ache, nausea, itching etc.
5 ( ) I have unbearable physical discomfort or symptoms, e.g. pain, ache, nausea, itching etc.

QUESTION 12. DEPRESSION
1 ( ) I do not feel at all sad, melancholic or depressed.
2 ( ) I feel slightly sad, melancholic or depressed.
3 ( ) I feel moderately sad, melancholic or depressed.
4 ( ) I feel very sad, melancholic or depressed.
5 ( ) I feel extremely sad, melancholic or depressed.

QUESTION 13. DISTRESS
1 ( ) I do not feel at all anxious, stressed or nervous.
2 ( ) I feel slightly anxious, stressed or nervous.
3 ( ) I feel moderately anxious, stressed or nervous.
4 ( ) I feel very anxious, stressed or nervous.
5 ( ) I feel extremely anxious, stressed or nervous.

QUESTION 14. VITALITY
1 ( ) I feel healthy and energetic.
2 ( ) I feel slightly weary, tired or feeble.
3 ( ) I feel moderately weary, tired or feeble.
4 ( ) I feel very weary, tired or feeble, almost exhausted.
5 ( ) I feel extremely weary, tired or feeble, totally exhausted.

QUESTION 15. SEXUAL ACTIVITY
1 ( ) My state of health has no adverse effect on my sexual activity.
2 ( ) My state of health has a slight effect on my sexual activity.
3 ( ) My state of health has a considerable effect on my sexual activity.
4 ( ) My state of health makes sexual activity almost impossible.
5 ( ) My state of health makes sexual activity impossible.
