# Supplementary material for: The effectiveness of a community-based, type 2 diabetes prevention programme on health-related quality of life. The DE-PLAN study
Source: PLoS One. 2019 Oct 11;14(10):e0221467. doi: 10.1371/journal.pone.0221467 (PMC6788719; doi:10.1371/journal.pone.0221467)
Supplement: S1 File — (ZIP) [file pone.0221467.s001.zip › basic_questionnaire.pdf]

DE-PLAN Basic Questionnaire

Date of visit (e.g. 260106)

d	d	m	m	y	y

Name: ____

Identity number: | | | | | | | | | | |

Date of birth (e.g. 231055)

d	d	m	m	y	y

Sex **1** **Male**

2 **Female**

SOCIODEMOGRAPHIC FACTORS

1. What is your marital status?

1. Married
2. Cohabiting
3. Single
4. Separated or divorced
5. Widow

2. What is your education?

1. Elementary school, basic education
2. Lower secondary education
3. Vocational school or relevant
4. Upper secondary education or high school
5. Non-university lower education
6. Non-university higher education
7. University education

3. How many years have you attended school and studied full time (basic levels included)?

__________ years (use numbers).

HEALTH STATUS AND FAMILY HISTORY OF DIABETES

4. Have you ever been diagnosed for diabetes?

1. No
2. Yes
3. Yes, gestational diabetes only

5. Has your biological father ever been diagnosed with diabetes?

1. No
2. Yes

6. Has your biological mother ever been diagnosed with diabetes?

1. No
2. Yes

7. How many siblings do you have? Mark 0, if you do not have siblings.

|__|__|

8. Has at least one of your siblings been diagnosed for diabetes?

1. No
2. Yes

9. Have you ever had any of the following diseases or abnormalities?

	No	Yes
High blood pressure, hypertension	1	2
Cardiac insufficiency	1	2
Angina pectoris, chest pain during exercise	1	2
Coronary artery disease	1	2
Myocardial infarction	1	2
Coronary (hearth) bypass surgery or angioplasty	1	2
Cerebral infarction, transient ischemic attack (TIA)	1	2
Claudication	1	2
High or heightened blood cholesterol level or dyslipidemia	1	2
Depression, other psychological illness	1	2
Physically handicapped	1	2
Other chronic disease,	1	2

specify? ______________________________

10. Please list all drugs you take regularly

____
____
____
____
____

11. Please list all vitamin and mineral preparations and health food shops' products you take regularly

____
____
____
____
____

SMOKING

12. Have you ever smoked?

1. No (You can skip questions 13-15)
2. Yes

13. Do you smoke now?

1. Not at all
2. Yes, occasionally
3. Yes, daily

14. If you smoked earlier but do not smoke now, when did you quit smoking?

1. 2 days – 1 month ago
2. 1 – 6 months ago
3. 6 – 12 months ago
4. 1 – 5 years ago
5. More than 10 years ago

15. If you smoke daily, how much you smoke per day (Use numbers)?

1. Cigarettes _____ per day
2. Pipes _______ per day
3. Cigars_________ per day

16. Have you planned to quit smoking?

1. No, I will not quit
2. Yes, I am planning to quit
3. Yes, I have tried to reduce / quit

EXERCISE AND OTHER PHYSICAL ACTIVITY

17. How demanding is your work physically? The activity at work is divided into four groups. If you do not work mark 1.

1. My work is <u>mainly done sitting down</u> and I do not walk much during my working hours (e.g. office work at a desk)
2. <u>I walk quite a lot in my work</u>, but I do not have to lift or carry heavy objects (e.g. office work which requires walking, store assistant)
3. I have <u>to walk and lift much</u> or to take the stairs or go uphill (e.g. a carpenter, heavier industrial work)
4. My work is <u>heavy manual labor</u> in which I have to lift or carry heavy objects, to dig, shovel or chop (e.g. forestry, heavy farm work, heavy construction or industrial work).

18. How many minutes do you walk, ride on a bicycle or otherwise exercise to get to work? (Please count in both traveling to and from work.)

1. I do not work or I use only a motorised vehicle
2. Less than 15 minutes daily
3. 15 - 29 minutes daily
4. 30 - 44 minutes daily
5. 45 - 59 minutes daily
6. Over an hour daily

19. How much do you exercise and work yourself physically in your spare time? If it varies much according to different seasons, mark the alternative which best describes the average situation.

1. In my spare time I read, watch TV, and work in the household with tasks which do not make me move much and which do not physically tax me.
2. In my spare time I walk, cycle or exercise otherwise at least 4 hours per week. This includes walking, fishing and hunting, light gardening etc. but excludes travel to work.
3. In my spare time I exercise to maintain my physical condition, e.g. running, jogging, skiing, gymnastics, swimming, playing football or other ball games or I do heavy gardening or the like for at least 3 hours per week.
4. In my spare time I exercise competatively several times a week by running, orienteering, skiing, swimming, playing ball games or other heavy sports.

20. How many times in a week do you exercise in your spare time so that you get at least mildly out of breath and sweaty? (if not at all, mark 0.)

__________ times a week

21. How long does your usual spare time physical activity take?

1. I do not exercise
2. Less than 15 minutes
3. 15 - 29 minutes
4. 30 - 59 minutes
5. One hour or longer

22. How many minutes do you daily walk, cycle or engage in a hobby in your spare time that requires moving about (yard work or gardening, fixing or cleaning the house)? Do not count in the activity needed at work (question 16), traveling to work (question 17) or spare time sports (questions 19 and 20).

1. Less than 15 minutes per day
2. 15-29 minutes per day
3. 30-44 minutes daily
4. 45-59 minutes daily
5. Over an hour per day

23. How do you consider your current physical condition?

1. Very good
2. Quite good
3. Fair
4. Quite bad
5. Very bad

24. Do you think that you exercise enough to maintain your physical condition or health?

1. No, I do not
2. Yes, I do

25. Have you increased your physical activity during the past year?

1. I have not / I don’t intend to increase in the near future
2. I have not, but I am going to increase in the near future
3. I have tried to increase
4. I have increased
5. I already get a lot of physical activity

NUTRITION

26. How many meals and snacks in all do you usually eat during weekday? Snack is e.g. fruit, chocolate bar, sandwich, juice, beer.

1. 1-2 meals and snacks
2. 3-4 meals and snacks
3. 5-6 meals and snacks
4. 7 or more

27. How many times a week do you eat the following dishes as a main course?

1. Fish dishes ______ times (e.g. baked fish, fried Baltic herrings, fish soup, herring)
2. Sausage meals ______ times (e.g. baked sausage, sausage soup, sausage stew)
3. Poultry or rabbit dishes______ times (e.g. grilled chicken, chicken fricassee, turkey)
4. Meat dishes______ times (e.g. meat soup, meatballs, pork chop, liver casserole, steak)
5. Vegetarian dishes______ times (e.g. vegetable soup, vegetable salad, spinach pancakes)

28. How many servings of fastfood do you eat? One serving is e.g. hamburger, meat pie, a piece of pizza, sandwich, a cup of potato chips.

1. Serving or more per day
2. 4-6 servings a week
3. 1-3 servings a week
4. 1-3 servings in a month
5. Less than 1 serving in a month or none

29. What type of cooking fat or oil is most often used in your household (Circle only one alternative.)

1. Mostly vegetable oil or soft margarine
2. Vegetable sterol margarine (e.g. Benecol, ProActive)
3. Butter or hard cooking margarine
4. No fat at all/ we do not cook

30. How often do you eat vegetables? One portion is e.g. 1 medium-sized carrot or 2 tomatoes, small salad or 2 tablespoons of cooked root vegetables.

1. 4 portions or more per day
2. 2-3 portions per day
3. 1 portion per day
4. 4-6 portions a week
5. 1-3 portions a week
6. Less than 1 portion a week or none

31. What kind of salad dressing do you usually use? (Circle only one alternative.)

1. Nothing
2. Vegetable oil-based dressing (olive oil, french dressing, mayonese etc.)
3. Juice-based dressing
4. A dressing based on cultured half cream or yogurt

32. How often do you eat fruit? One portion is an apple, 2 mandarins, 5 strawberries etc. medium-sized fruit or 2 deciliters of berries or fruit salad.

1. 4 portions or more per day
2. 2–3 portions per day
3. 1 portion per day
4. 4–6 portions a week
5. 1–3 portions a week
6. Less than 1 portion a week or none

33. How many servings of <u>milk or liquid milk products</u> do you have <u>per day?</u> 1 serving = 200 mls= 1/3 pint

1. ______ servings of milk products with < 1 % fat (skimmed milk or fat-free yogurt)
2. ______ servings of milk products with 1-<2 % fat (semi-skimmed milk)
3. ______ servings of milk products with 2-3 % fat (full fat milk or regular yogurt)
4. ______ servings of milk products with >3 % fat or more
5. I do not usually have milk products

34. How much do you <u>usually</u> eat bread, other cereals, potatoes, and legumes <u>per day?</u> A slice of bread is about 30 gram.

1. _______ slices of rye- or crispbread (>10 g fibre/100g)
2. _______ slices of graham- or mixed grain bread or roll (4-10 g fibre/100g)
3. _______ slices of white bread or roll (< 4 g fibre/100g)
4. _______ deciliters of porridge (e.g. rye-, oat- or wheat flake porridge)
5. _______ deciliters of low-fibre breakfast cereals (e.g. corn flakes or rice crispies)
6. _______ deciliters of muesli or high-fibre breakfast cereals
7. _______ slices of sweet bread
8. _______ deciliters of pasta or rice
9. _______ deciliters of potatoes (baked, boiled, gnocchi, gateau??)
10. _______ deciliters of legumes (lentils, chick-peas, peas)

35. How often do you eat nuts or seeds? One portion is about 1 tablespoon (15g)

1. 2 portions or more per day
2. 1 portion per day
3. 4–6 portions a week
4. 1–3 portions a week
5. Less than 1 portion a week or none

36. What kind of spread do you <u>usually</u> use on your bread? (Circle only one alternative.)

1. Reduced-fat margarine (28-60% fat) (…)
2. Soft margarine with 70-80% fat (…)
3. Vegetable sterol margarine (e.g. Benecol, ProActive)
4. Butter-vegetable oil mixture (…)
5. Butter
6. Nothing

37. How much do you usually eat cheese per day? A slice of cheese is about 10 gram.

1. _______ slices of cheese with < 20% fat (…)
2. _______ slices of cheese with > 20% fat (…)
3. _______ slices of cheese with vegetable fat (…)
4. Less than a slice per day or none

38. How much do you usually use cold cuts per day? A slice is about 10 gram.

1. ________ slices of cold cuts with <10% fat (…)
2. ________ slices of sausages or cold cuts with >10% fat (…)
3. ________ pieces of frankfurter (35g) etc. (…)
4. Less than a slice per day or none

39. How much do you eat sweet patisseries, ice cream, puddings or chocolate? One portion is e.g. a piece of pie or cake, a small doughnut or Danish pastry, 3-4 cookies, ice cream cornet, chocolate bar.

1. 2 portions or more per day
2. 1 portion per day
3. 4–6 portions a week
4. 1–3 portions a week
5. Less than 1 portion a week or none

40. How much do you eat sugar, honey or sweets? On portion is e.g. 2 teaspoons of sugar or honey, 3 sugar lumps, 5 sweets.

1. 2 portions or more per day
2. 1 portion per day
3. 4–6 portions a week
4. 1–3 portions a week
5. Less than 1 portion a week or none

41. How much do you usually drink the following beverages a week? Mark 0, if you do not drink certain drink at all.

1. ________ cups of regular tea (1 cup = 2 deciliters or 200 mls)
2. ________ cups of decaffeinated tea (1 cup = 2 deciliters)

3. ________ cups of regular coffee (1 cup = 1 deciliter)
4. ________ cups of decaffeinated coffee (1 cup = 1 deciliter)

5. ________ bottles of soft drink with sugar (1 bottle = 1/3 liters)
6. ________ bottles of sugar-free soft drink (e.g. Coca Cola Light)
7. ________ glasses of fruit juice (1 glass = about 2 deciliters)
8. ________ glasses of sugar-sweetened juice

9. ________ bottles of beer, cider etc. with alcohol content <4,7 %
10. ________ bottles of strong beer, cider etc. with alcohol content >4,7 %
11. ________ glasses of wine (1 glass = 12 cl)
12. ________ portions of spirits (e.g. vodka, whisky, gin, cognac, liquers etc. 1 portion= 4 cl)

42. Have you decreased the amount of fat in your diet <u>during the past year</u>? For example changed whole milk to skimmed milk, decreased the amount of spread on your bread or tried to choose foods with low-fat.

1. I have not and I don't intend to decrease in the near future
2. I have not, but I am going to decrease in the near future
3. I have tried to decrease
4. I have decreased
5. My diet is already low-fat

43. Have you changed the quality of fat used in your household <u>during the past year</u> (saturated fat→ unsaturated fat)? For example started to use oil instead of hard margarine when cooking, started to use low-fat margarine spread instead of butter on bread or increased the consumption of fish?

1. I have not and I don't intend to change in the near future
2. I have not, but I am going to change in the near future
3. I have tried to change
4. I have changed
5. I already use mainly unsaturated fat instead of saturated fat

44. Have you increased consumption of vegetables, fruits and berries <u>during the past year</u>?

1. I have not and I don't intend to increase in the near future
2. I have not, but I am going to increase in the near future
3. I have tried to increase
4. I have increased
5. I already use a lot of vegetables, fruits and berries

45. Have you decreased consumption of alcohol <u>during the past year</u>?

1. I have not and I don't intend to decrease in the near future
2. I have not, but I am going to decrease in the near future
3. I have tried to decrease
4. I have decreased
5. I already use alcohol only moderately / I do not use at all

46. Have you lost weight <u>during the past year</u>?

1. I have not and I don't intend to lose weight in the near future
2. I have not, but I am going to lose weight in the near future
3. I have tried to lose weight
4. I have lost weight
5. I am already pleased with my weight
