# Supplementary material for: The effectiveness of a community-based, type 2 diabetes prevention programme on health-related quality of life. The DE-PLAN study
Source: PLoS One. 2019 Oct 11;14(10):e0221467. doi: 10.1371/journal.pone.0221467 (PMC6788719; doi:10.1371/journal.pone.0221467)
Supplement: S1 File — (ZIP) [file pone.0221467.s001.zip › clinical_data_collection_form.pdf]

DE-PLAN Clinical Data Collection Form

Date of visit (e.g. 260106)

d	d	m	m	y	y

Name: ______________________________

Identity number: | | | | | | | | | | |

Date of birth (e.g. 231055)

d	d	m	m	y	y

Sex **1** **Male**
2 **Female**

1. Diabetes Risk Test, total score: | | |

2. Medicines in use

	**No**	**Yes**
Drugs for diabetes	**1**	**2**
Acetylsalicylic acid (ASA)	**1**	**2**
Drugs to lower lipid level	**1**	**2**
Drugs for high blood pressure	**1**	**2**
Drugs for coronary artery disease	**1**	**2**
Drugs for weight loss	**1**	**2**
Nicotine replacement therapy	**1**	**2**
Antidepressant	**1**	**2**

3. Diseases and health problems (diagnosed earlier or during this visit)

	**No**	**Yes**
Coronary artery disease	**1**	**2**
Cerebral artery disease	**1**	**2**
Peripheric artery disease	**1**	**2**
Retinopathy	**1**	**2**
Neuropathy	**1**	**2**
Nephropathy	**1**	**2**
Foot problem	**1**	**2**

4. Height (cm)

|___|___|___| **cm**

5. Weight (kg)

|___|___|___| **,** |___| **kg**

6. BMI (kg/m^2)

|___|___| **,** |___|

7. Waist circumference (cm)

|___|___|___| **cm**

8. Blood pressure (mmHg)

First measurement (systolic/diastolic)

|___|___|___| **/** |___|___|___|

Second measurement (systolic/diastolic)

|___|___|___| **/** |___|___|___|

9. Basic questionnaire has been filled in and checked

1. Yes
2. No

10. This form filled by

1. Nurse
2. Physician
3. Someone else, who? ____________________________________

11. GLUCOSE MEASUREMENTS

Date (e.g. 260106)

d d m m y y

[ ][ ][ ][ ][ ][ ]

Type of blood sample

1 Venous
2 Capillary

Glucose analysed from

1 Plasma
2 Whole blood
3 Capillary blood
4 Serum

Glucose fasting value (mmol/l)

[ ][ ] , [ ]

Glucose 2-h value (mmol/l)

[ ][ ] , [ ]

Classification of glucose metabolism

1 Normal
2 IFG (impaired fasting glucose) only
3 IGT (impaired glucose tolerance)
4 Diabetes

12. LIPID MEASUREMENTS

Date (e.g. 260106)

d d m m y y

[ ][ ][ ][ ][ ][ ]

Lipids analysed from

1 Plasma
2 Serum

Overnight fast

1 Yes
2 No

Cholesterol (mmol/l)

[ ][ ] , [ ]

HDL-Cholesterol (mmol/l)

[ ][ ] , [ ]

Triglyserides (mmol/l)

[ ][ ] , [ ]
