# Supplementary material for: The effectiveness of a community-based, type 2 diabetes prevention programme on health-related quality of life. The DE-PLAN study
Source: PLoS One. 2019 Oct 11;14(10):e0221467. doi: 10.1371/journal.pone.0221467 (PMC6788719; doi:10.1371/journal.pone.0221467)
Supplement: S1 File — (ZIP) [file pone.0221467.s001.zip › intervention_form.pdf]

DE-PLAN Intervention Form

Date of visit (e.g. 250106)

d	d	m	m	y	y

Name: ______________________________

Identity number:

Sex **1 Male**
2 Female

Date of birth (e.g. 231055)

d	d	m	m	y	y

**Date of visit**						**Type of visit**		**Advisor**	**Focus**
d	d	m	m	y	y	Individual	Group	1. Nurse<br>2. Physician<br>3. Physiotherapist<br>4. Dietitian<br>5. Psychologist<br>6. Other	1. Diet / weight loss<br>2. Exercise<br>3. Smoking withdrawal<br>4. Other
						1	2		
						1	2		
						1	2		
						1	2		
						1	2		
						1	2		
						1	2		
						1	2		
						1	2		
						1	2		
						1	2		
						1	2		
						1	2		
