# Supplementary material for: The effectiveness of a community-based, type 2 diabetes prevention programme on health-related quality of life. The DE-PLAN study
Source: PLoS One. 2019 Oct 11;14(10):e0221467. doi: 10.1371/journal.pone.0221467 (PMC6788719; doi:10.1371/journal.pone.0221467)
Supplement: S1 Table — (DOC) [file pone.0221467.s003.doc]

Supplementary material Table : Comparison of baseline characteristics of participants with full set of data vs. participants without.

| **Variable** | **Participants with full set of HRQQL data** | **Participants without full set of HRQQL data** | **Total** | P-value |
| --- | --- | --- | --- | --- |
| Number of participants | 786 | 293 | 1079 |  |
| Males/Females (n, %) | 259 (33%) /527 (67%) | 83(35.3%) /210 (64.7%) | 342 (31.5%) /737 (68.3%) | 0.13 |
| Age (years) | 59.7 ±9.4 | 57.6 ±11.1 | 59 ±9.8 | **0.005** |
| Current smokers (n, %) | 328 (41.7%) | 164 (37.8%) | 492 (45.6%) | 0.7 |
| Weight (kg) | 82.7 ±14.3 | 84.9 ±16.25 | 83.4 ±14 | 0.058 |
| BMI (kg/m^2^) | 31.5 ±4.5 | 32.1 ±5.4 | 31.7 ±4.8 | 0.13 |
| Participants with pre-diabetes (n, %) | 350 (44.5%) | 137 (46.8%) | 487(45.1%) | 0.8 |
| Waist circumference (cm) | 100.4 ±10.5 | 101.5 ±11.7 | 100.9 ±10.1 | 0.2 |
| Male (n=345) | 104. 5 ±9.5 | 106.5±11 | 103. 5 ±10 | 0.1 |
| Female (n=740) | 98.4 ±10.4 | 99.6 ±11.3 | 98.9 ±10.9 | 0.2 |
| Systolic blood pressure (mmHg) | 136.2 ±16.9 | 133.7 ±17.1 | 135.6 ±17 | **0.024** |
| Diastolic blood pressure (mmHg) | 81.6 ±10.4 | 81.6 ±11.7 | 81.7 ±10.7 | 0.8 |
| Fasting Glucose (mmol/l) | 5.4 ±0.7 | 4.9 ±1 | 5.0 ±1 | 0.2 |
| Total cholesterol (mmol/l) | 5.5 ±1 | 5.6 ±0.9 | 5.6 ±1 | 0.7 |
| HDL cholesterol (mmol/l) | 1.4 ±0.35 | 1.4 ±0.4 | 1.4 ±0.4 | **0.05** |
| Triglycerides (mmol/l) | 1.5 ±0.9 | 1.1 ±1 | 1.6 ±1.1 | 0.9 |
| LDL cholesterol (mmol/l) | 3.4 ±0.93 | 3.5 ±0.9 | 3.4 ±0.95 | 0.2 |
| D15 score at baseline | 0.88±0.9 | 0.87±0.9 |  | **0.02** |

Mean ±SD or n (%). BMI, body mass index; HDL, high-density lipoprotein; LDL, low-density lipoprotein
